# Aphid-Triggered Changes in Oxidative Damage Markers of Nucleic Acids, Proteins, and Lipids in Maize (*Zea mays* L.) Seedlings

**DOI:** 10.3390/ijms20153742

**Published:** 2019-07-31

**Authors:** Hubert Sytykiewicz, Iwona Łukasik, Sylwia Goławska, Grzegorz Chrzanowski

**Affiliations:** 1 Department of Biochemistry and Molecular Biology, Siedlce University of Natural Sciences and Humanities, 14 Prusa St., 08-110 Siedlce, Poland; 2 Department of Molecular Biotechnology, University of Rzeszow, 1 Pigonia St., 35-310 Rzeszow, Poland

**Keywords:** maize, *Rhopalosiphum padi*, oxidation of nucleic acids, carbonyl groups, thiols, malondialdehyde, electrolyte leakage

## Abstract

Prior experiments illustrated reactive oxygen species (ROS) overproduction in maize plants infested with bird-cherry-oat (*Rhopalosiphum padi* L.) aphids. However, there is no available data unveiling the impact of aphids feeding on oxidative damages of crucial macromolecules in maize tissues. Therefore, the purpose of the current study was to evaluate the scale of oxidative damages of genomic DNA, total RNA and mRNA, proteins, and lipids in seedling leaves of two maize genotypes (Złota Karłowa and Waza cvs—susceptible and relatively resistant to the aphids, respectively). The content of oxidized guanosine residues (8-hydroxy-2′-deoxyguanosine; 8-OHdG) in genomic DNA, 8-hydroxyguanosine (8-OHG) in RNA molecules, protein carbonyl groups, total thiols (T-SH), protein-bound thiols (PB-SH), non-protein thiols (NP-SH), malondialdehyde (MDA) and electrolyte leakage (EL) levels in maze plants were determined. In addition, the electrical penetration graphs (EPG) technique was used to monitor and the aphid stylet positioning and feeding modes in the hosts. Maize seedlings were infested with 0 (control), 30 or 60 *R. padi* adult apterae per plant. Substantial increases in the levels of RNA, protein and lipid oxidation markers in response to aphid herbivory, but no significant oxidative damages of genomic DNA, were found. Alterations in the studied parameters were dependent on maize genotype, insect abundance and infestation time.

## 1. Introduction

Maize (*Zea mays* L.; Poaceae) has emerged as an important crop of increasing significance in several agronomic, pharmaceutical, nutraceutical and industrial areas [1,2]. In addition, it is a commonly used model plant in a variety of biochemical, molecular and genetic experiments [3,4]. Aphids are among the most detrimental insect pests, profoundly limiting yields and deteriorating quality of maize tissues [5,6,7]. In Poland, bird cherry-oat aphids (*Rhopalosiphum padi* L.) reach high densities on maize from May to October, and can generate up to twelve generations [8]. Previously, Sytykiewicz et al. [9] have classified fifteen maize cultivars with varying levels of resistance to *R. padi* adults. Additionally, our research team documented an imbalance towards the pro-oxidative side of the redox homeostasis in maize plants infested with *R. padi* [4,5,6,9,10,11]. However, until now, there is no available information regarding the effect of aphid colonization on oxidative damages of nucleic acids (genomic DNA, total RNA, mRNA) in host plants. In addition, intervarietal differences in level of biochemical markers of oxidative damages of proteins and lipids in aphid-stressed plants are highly unclear.

The study of aphid feeding behaviour provides comprehensive information on the sophisticated and complex relationships between the insect and its host plant. A detailed description of the feeding behaviour of the aphids on different plant species is quite difficult because they have to penetrate through several tissues to reach the target feeding site, preferably the phloem sap. Activity of the stylets, salivation or food intake cannot be observed externally and requires an indirect visualization technique, such as the electrical penetration graph (EPG). The parameters describing aphid behaviour during probing (the aphid stylet penetration in plant tissues) and feeding (phloem sap uptake), such as total time of probing, number of probes, duration of phloem sap ingestion, and duration of the sap ingestion from one sieve element are common indicators of either plant suitability or interference with probing by chemical or physical factors in particular plant tissues [12,13]. Exploitation of hosts by removing large amounts of phloem sap during aphid herbivory leads to substantial disturbances in the content of numerous chemical compounds within plant tissues. Quantity and specific composition of several constituents in the phloem sap are major factors affecting aphid growth and development [12,13,14].

We hypothesized that aphid-triggered oxidative stress in foliar tissues of maize seedlings may lead to severe damage to the plasmalemma and crucial cellular biomolecules such asnucleic acids, proteins, and lipids. Biological consequences of excessive generation of reactive oxygen species (ROS) in aphid-attacked maize cultivars displaying distinct resistance degrees still remains unclear. In order to test our hypothesis, a number of indicators of aphid-induced oxidative damages in the insect-treated maize varieties differing in the resistance levels were analysed. Specifically, we: (1) measured oxidative stress-induced damages in genomic DNA molecules—quantitation of 8-hydroxy-2′-deoxyguanosine (8-OHdG); (2) assessed oxidative damages of total RNA and mRNA—measurement of oxidized guanosine residues (8-hydroxyguanosine; 8-OHG); (3) assessed protein oxidation level by estimation of carbonyl groups’ content in proteins; (4) determined total thiols (T-SH), protein-bound thiols (PB-SH) and non-protein thiols (NP-SH)—biomarkers of the oxidative stress; (5) measured malondialdehyde (MDA) content indicating degree of lipid peroxidation; (6) evaluated plasma membrane stability, based on the electrical conductivity measurement. In addition, we applied the electrical penetration graphs (EPG) technique to precisely monitoring aphid stylet positioning and feeding modes in tissues/cells of susceptible and relatively resistant maize genotypes (Złota Karłowa and Waza cvs, respectively).

## 2. Results

### 2.1. Impact of Aphid Herbivory on the Content of Oxidized Guanosine Residues in Genomic DNA and RNA Samples Derived from Maize Seedlings

**Figure 1:**
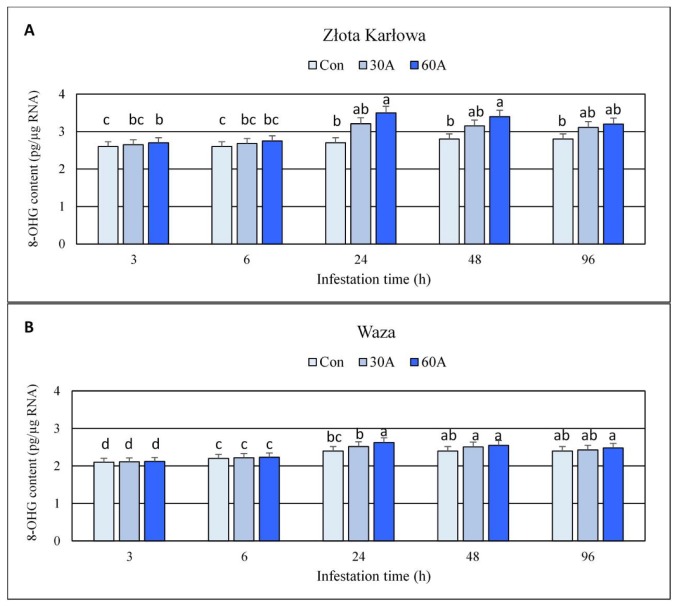
Effect of *R. padi* herbivory on the content of 8-hydroxyguanosine (8-OHG) in total RNA in maize seedlings. (**A**)—Złota Karłowa cultivar (susceptible), (**B**)—Waza cultivar (relatively resistant). Con—uninfested plants, 30A and 60A—30 and 60 aphids per plant, respectively. Different letters above bars denote significant differences between the means of treated (30A and 60A) and control plants of each genotype (Tukey’s test; *p* < 0.01).

**Figure 2:**
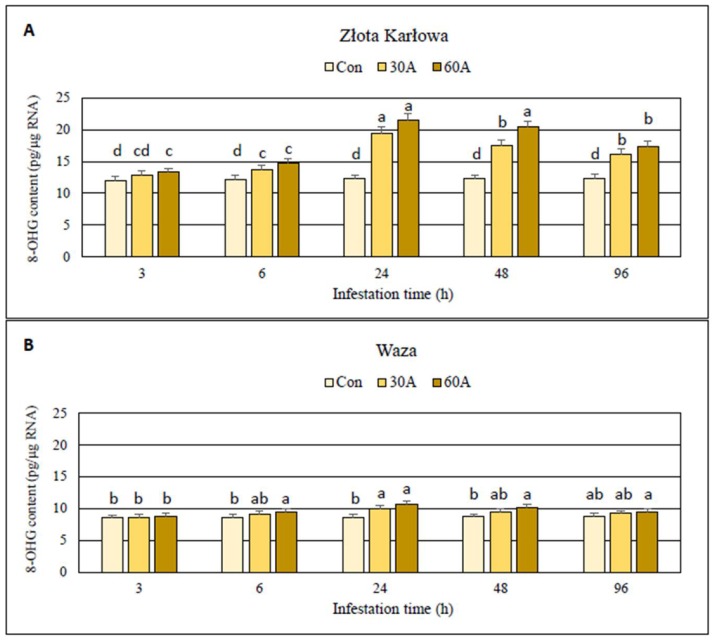
Accumulation of 8-hydroxyguanosine (8-OHG) amount in mRNA in aphid-treated maize seedlings. (**A**)—Złota Karłowa cultivar (susceptible), (**B**)—Waza cultivar (relatively resistant). Con—uninfested plants, 30A and 60A—30 and 60 aphids per plant, respectively. Different letters above bars denote significant differences between the means of treated (30A and 60A) and control plants of each genotype (Tukey’s test; *p* < 0.01).

The experiments revealed insignificant increases (up to 4%; *p* > 0.05) in the content of 8-hydroxy-2′-deoxyguanosine (8-OHdG) in genomic DNA in the aphid-infested seedlings of Złota Karłowa and Waza cultivars, compared to the insect-free controls (Table S1). Importantly, *R. padi* infestation considerably increased the content of oxidized guanosine residues (8-hydroxyguanosine; 8-OHG) in both total RNA and mRNA in foliar tissues of the two maize cultivars, in comparison with the non-infested controls (Figure 1 and Figure 2; Table S1). Further, higher oxidation level occurred in mRNA in comparison with the total RNA in aphid-stressed seedlings of both maize cultivars. In addition, the highest increment in 8-OHG level was noted after 24-h of the aphid feeding. At this time-point, susceptible Złota Karłowa seedlings had approximately 2-fold higher increases in 8-OHG content in mRNA and about 20% higher oxidation level in total RNA, respectively, compared to resistant Waza plants (Figure 2; Table S1). Moreover, 60 *R. padi* females per plant caused greater increases in the content of 8-OHG in both analysed RNA fractions in both maize cultivars, compared to the lower level of infestation (30 aphids per plant).

### 2.2. Protein-Bound Carbonyls (PC) Content in Aphid-Challenged Maize Plants

**Figure 3:**
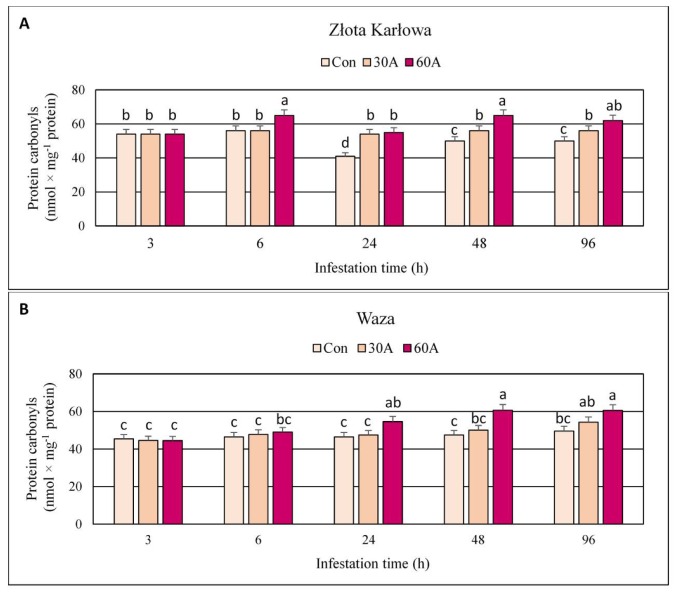
Impact of the bird cherry-oat aphid feeding on the content of protein-bound carbonyls in maize plants. (**A**)—Złota Karłowa cultivar (susceptible), (**B**)—Waza cultivar (relatively resistant). Con—control (non-infested) plants; 30A and 60A—30 and 60 aphids per plant, respectively. Different letters above bars denote significant differences between the means of treated (30A and 60A) and control plants of each genotype (post-hoc Tukey’s test; *p* < 0.01).

Wingless females of the bird cherry-oat aphid affected the accumulation of protein-bound carbonyls (PC) in the seedlings of both maize cultivars (2–35% increase in comparison to the aphid-free plants, depending on insect densities and maize cultivars) (Figure 3; Table S2). Furthermore, higher increases in PC amount were noted in tissues of susceptible Złota Karłowa cv. than in seedlings of more resistant Waza cv. (10–35% and 2–28% increases, respectively). However, at 3 h post infestation (hpi) the aphid herbivory did not stimulate any changes in the content of PC in both maize genotypes. In addition, at 6 hpi, only the highest densities of the aphids (60 females per plant) evoked significant increases in PC level in Złota Karłowa and Waza seedlings. Similar trend occurred at 24 hpi in Waza plants infested with *R. padi*. Overall, greater increases in the content of protein carbonyl groups were found in the maize seedlings exposed to 60 aphids per plant, compared to the lower infestation level (30 aphids per plant). From 6 to 96 hpi, the aphid-free (control) plants exhibited slight increases in PC amount in seedlings of Waza cv., and minor alternations in Złota Karłowa plants.

### 2.3. Effect of R. padi Herbivory on the Content of Protein Thiols (PT) in Maize Tissues

**Figure 4:**
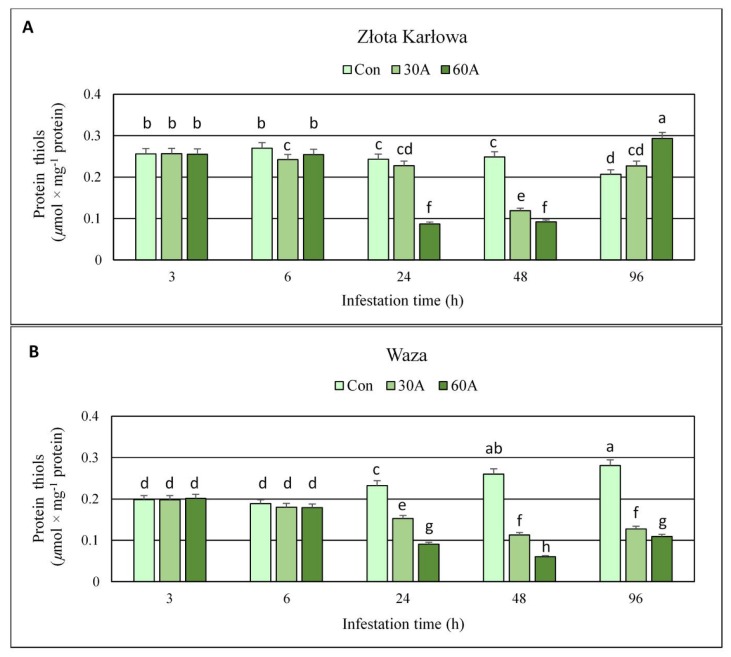
Aphid-induced changes in levels of protein thiols in maize plants. (**A**)—Złota Karłowa cultivar (susceptible), (**B**)—Waza cultivar (relatively resistant). Con—control (non-infested) plants; 30A and 60A—30 and 60 aphids per plant, respectively. Different letters above bars denote significant differences between the means of treated (30A and 60A) and control plants of each genotype (*post-hoc* Tukey’s test; *p* < 0.01).

Protein thiols (PT) amounts remained stable after 3-h aphid feeding on seedlings of both maize cultivars (Figure 4, Table S2). Prolonged exposure to insects (6–48 hpi) was linked to depletion in PT levels in foliar tissues of maize genotypes (10–64% and 5–77% declines in Złota Karłowa and Waza seedlings, respectively). After 96 hpi, susceptible Złota Karłowa plants infested with 30 or 60 aphids characterized with increased PT content (10% and 42%, respectively), whereas the opposite trend was noted in the resistant Waza cultivar (54% and 61% decrements, respectively). Stronger effect of higher number of aphids (60 per plant) on the content of PT, compared to lower aphid abundance (30 per plant) was also observed. Furthermore, between 6 and 96 hpi, gradual increases in PT level in the aphid-free seedlings of Waza cv. and its slight changes in Złota Karłowa genotype were noted.

### 2.4. Influence of Aphid Feeding on the Content of Non-Protein Thiols (NPT) in Z. mays Seedlings

**Figure 5:**
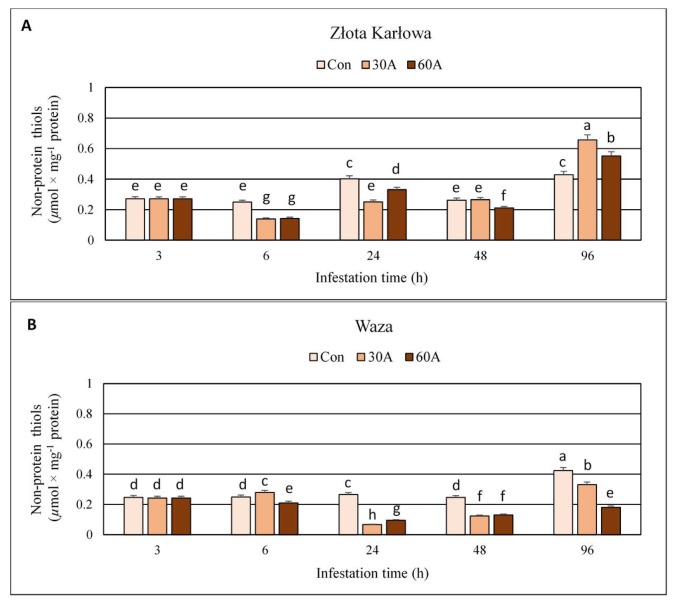
Impact of the bird cherry-oat aphid herbivory on the content of non-protein thiols in maize seedlings. (**A**)—Złota Karłowa cultivar (susceptible), (**B**)—Waza cultivar (relatively resistant). Con—control (non-infested) plants; 30A and 60A—30 and 60 aphids per plant, respectively. Different letters above bars denote significant differences between the means of treated (30A and 60A) and control plants of each genotype (*post-hoc* Tukey’s test; *p* < 0.01).

Three-hour long herbivory by *R. padi* did not affect the content of non-protein thiols (NPT) in the seedlings of the two maize varieties (Waza and Złota Karłowa) (Figure 5; Table S2). From 6 to 48 hpi, insect feeding resulted in 18–44% decreases in NPT level in Złota Karłowa (susceptible) plants; however, at 48 hpi, infestation with 30 aphids per plant did not induce any changes in the quantified thiols, compared to the non-infested control. On the contrary, after 96 hpi, NPT content increased by 29–53% in aphid-stressed Złota Karłowa seedlings, compared to the aphid-free plants of the same variety. In addition, at 6 hpi, the amount of NPT increased (12%) in relatively resistant Waza cultivar infested with 30 aphids per plant, but all the other aphid treatments (up to 96 hpi) resulted in significant decreases (16–75%) in levels of the analysed thiols. Moreover, the content of NPT in all plants free of the aphids fluctuated significantly across the timespan of the experiment.

### 2.5. Aphid-Induced Changes in the Content of Total Thiols (TT) in Maize Seedlings

**Figure 6:**
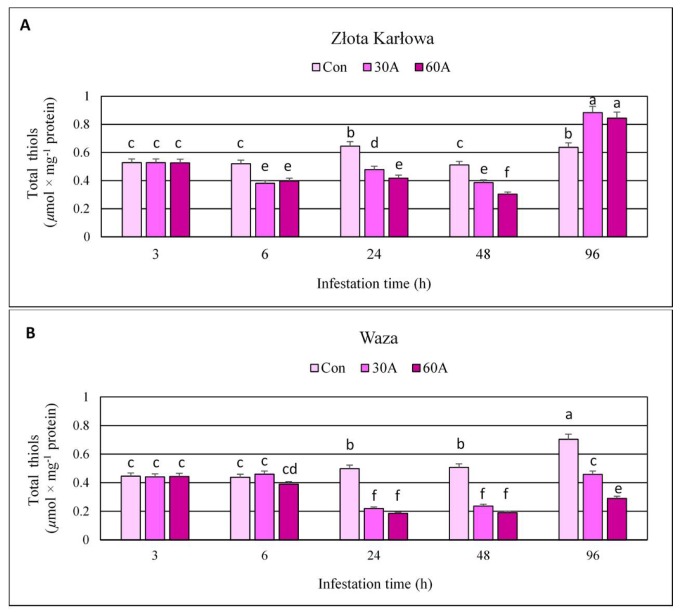
Influence of *R. padi* feeding on the content of total thiols in maize seedlings. (**A**)—Złota Karłowa cultivar (susceptible), (**B**)—Waza cultivar (relatively resistant). Con—uninfested plants, 30A and 60A—30 and 60 aphids per plant, respectively. Different letters above bars denote significant differences between the means of treated (30A and 60A) and control plants of each genotype (*post-hoc* Tukey’s test; *p* < 0.01).

Short-time (3 h) infestation of Waza and Złota Karłowa maize seedlings with *R. padi* did not evoke significant alternations in the content of total thiols (Figure 6, Table S2). Only at three time-points, TT level increased (at 3 hpi—about 5% increase in Waza seedlings infested with 30 aphids, and after 96 hpi—33% and 39% increases in Złota Karłowa seedlings infested with 30 and 60 aphids per plant, respectively, in relation to the uninfested controls). All the other *R. padi* treatments led to a decline in the content of TT in the tested maize seedlings. However, higher decreases were observed in more resistant Waza seedlings (1–63%), compared to Złota Karłowa plants (0.3–41%). In general, greater decreases in the content of TT occurred in the maize seedlings exposed to higher abundance of aphids (60 per plant), compared to the lower infestation level (30 females per plant). In addition, significant increases in TT amount in non-infested Waza seedlings were found, whereas the level of the quantified thiols slightly fluctuated in Złota Karłowa plants between 6 and 96 hpi.

### 2.6. Effect of Insect Feeding on the Content of Malondialdehyde (MDA) and Plasma Membrane Stability in Maize Plants

**Figure 7:**
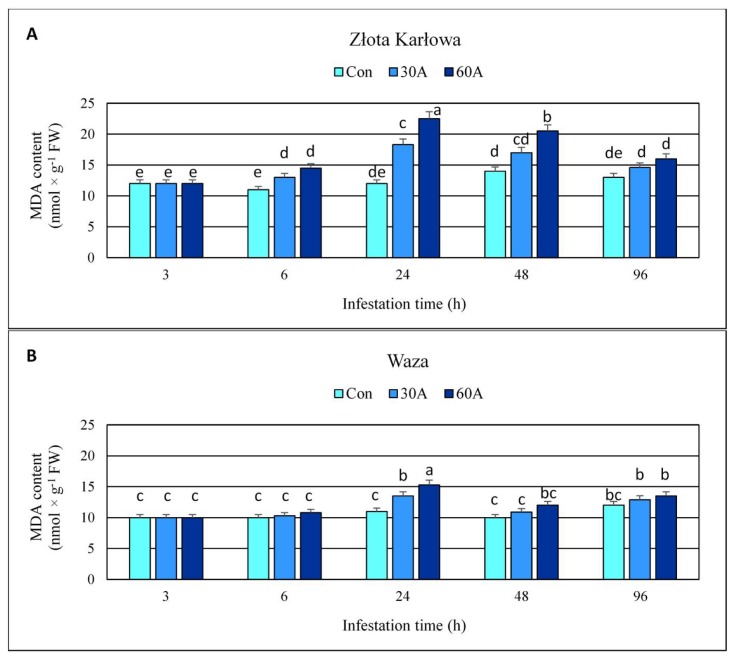
Effect of the bird cherry-oat aphid herbivory on the amount of malondialdehyde (MDA) in maize plants. (**A**)—Złota Karłowa cultivar (susceptible), (**B**)—Waza cultivar (relatively resistant). Con—uninfested plants, 30A and 60A—30 and 60 aphids per plant, respectively. Different letters above bars denote significant differences between the means of treated (30A and 60A) and control plants of each genotype (Tukey’s test; *p* < 0.01).

After 3-h aphid herbivory, the content of MDA remained unaltered in the seedling leaves of both maize cultivars, in comparison to the uninfested control plants (Figure 7; Table S1). However, all the other tested time periods of the aphid feeding resulted in augmented accumulation of MDA in maize tissues (13–85% and 2–39% increases in Złota Karłowa and Waza seedlings, respectively), compared to the controls. In addition, the maximum increases in MDA content were recorded at 24 hpi, and reached approximately 2-fold higher levels in Złota Karłowa plants, compared to Waza seedlings. Importantly, the highest density of aphids (60 *R. padi* apterae per plant) caused 5–32% greater elevations in MDA content in the maize cultivars across the timepoints, compared to the plants exposed to 30 aphids.

**Figure 8:**
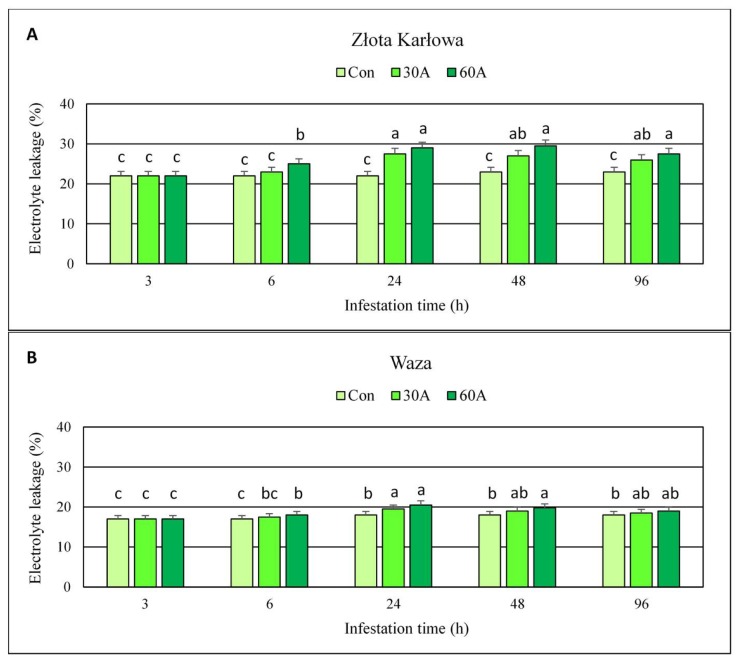
Changes in the electrolyte leakage (EL) levels in *R. padi*-stressed maize seedlings. (**A**)—Złota Karłowa cultivar (susceptible), (**B**)—Waza cultivar (relatively resistant). Con—uninfested plants, 30A and 60A—30 and 60 aphids per plant, respectively. Different letters above bars denote significant differences between the means of treated (30A and 60A) and control plants of each genotype (Tukey’s test; *p* < 0.01).

Bird cherry-oat aphid feeding led to significant increases (up to 30%, compared to the aphid-free control) in electrolyte leakage (EL) level in the maize seedlings of both Złota Karłowa and Waza genotypes (Figure 8; Table S1). The exception was a 3-h period of *R. padi* feeding, during which no effect on the quantified parameter in maize plants was observed. Additionally, the highest increases in EL values in Złota Karłowa and Waza seedlings at 24 hpi were noted (24–30% and 8–12% elevations, respectively, compared to the control). Furthermore, aphid-stressed Złota Karłowa seedlings had up to 3-fold higher levels of EL percentage than Waza plants (4–30% and 2–12% increases, respectively, in comparison with the control). Both tested levels of insect herbivory (30 and 60 females per plant) resulted in similar increases of EL in the examined maize seedlings (2–24% and 5–30%, respectively).

### 2.7. Electrical Penetration Graph (EPG) Recordings of Feeding Behavior of R. padi

**Figure 9:**
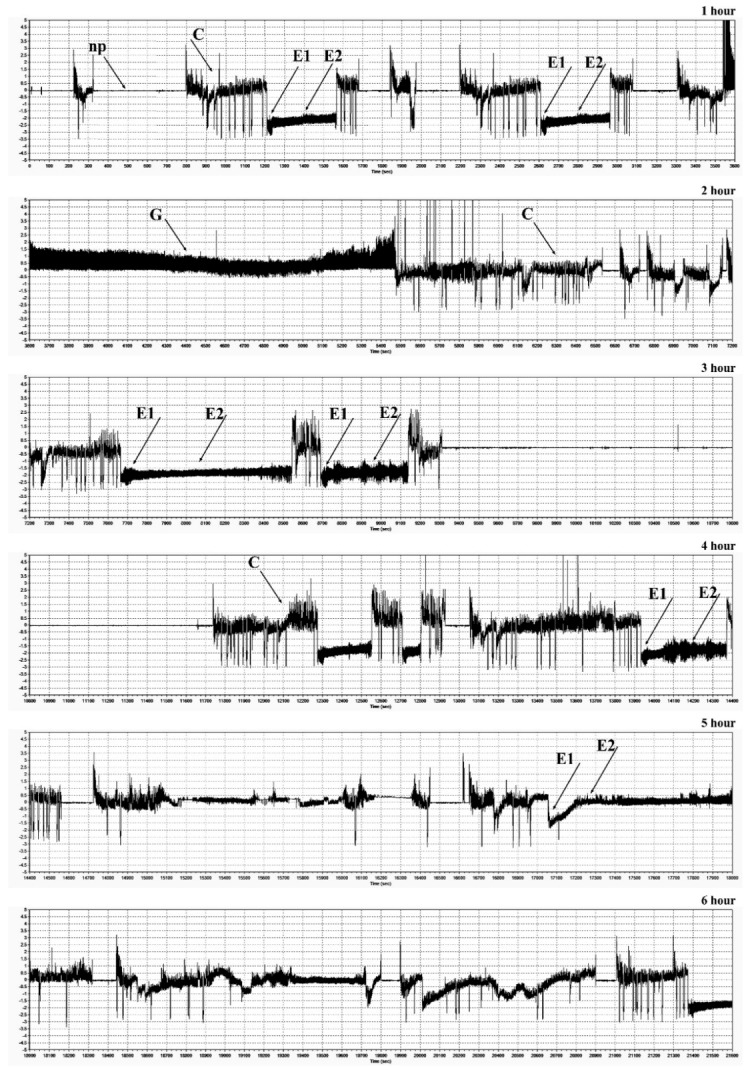
EPG recordings of stylet activities of *R. padi* within seedling leaves of maize (susceptible Złota Karłowa cultivar). Horizontal axis—time (s); vertical axis—amplitude (mV). Np—non-probing; (**C**)—transition of the insect stylets through epidermis and mesophyll cells; (**E1**)—salivation into sieve elements; (**E2**)—phloem sap ingestion; (**G**)—xylem sap uptake.

**Figure 10:**
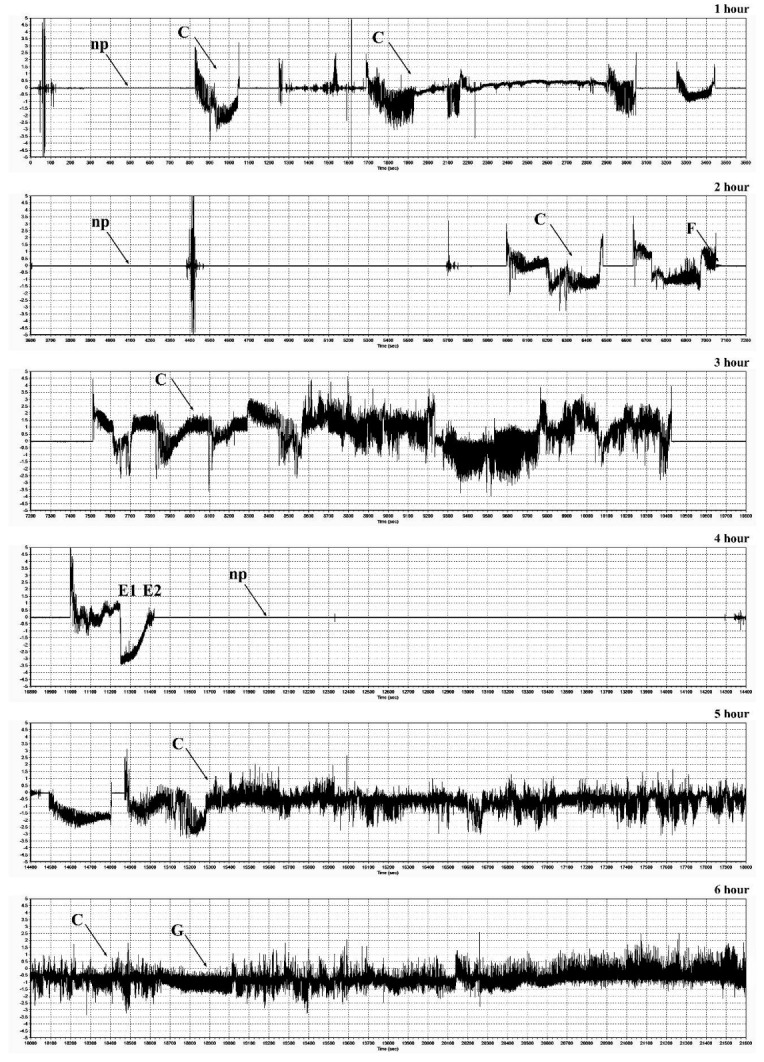
EPG recordings of stylet activities of *R. padi* within seedling leaves of maize (relatively resistant Waza cultivar). Horizontal axis—time (s); vertical axis—amplitude (mV). Np—non-probing; (**C**)—transition of the insect stylets through epidermis and mesophyll cells; (**E1**)—salivation into sieve elements; (**E2**)—phloem sap ingestion; (**F**)—mechanical movements of stylets associated with difficulties during tissue puncturing; (**G**)—xylem sap uptake.

EPG recordings performed during infestation of maize plants (Waza and Złota Karłowa cvs) with females of the bird cherry-oat aphid, demonstrated the occurrence of major feeding waveforms: C model (indicated transition of the insect stylets through epidermis and mesophyll cells), E1 and E2 models (in which sieve elements were pierced, watery saliva was injected into them, and next, phloem sap was ingested), G model (uptake of xylem sap), and F model (associated with difficulties in stylet penetrations in tissues of the host plant) (Figure 9 and Figure 10; Table 1). There were significant differences in the feeding behavior of *R. padi* females fed on the susceptible and resistant maize cultivars. Generally, the total and mean probing times were considerably longer on the resistant cultivar than on the susceptible one. The aphids that fed on the resistant seedlings exhibited a prolonged time of penetration of the epidermis and mesophyll as well as time to first probing, in comparison with the susceptible cultivar. Insects feeding on the resistant plants also showed a reduction of phloem sap ingestion. Duration time of the first C pattern was 6.54 and 18.66 min on the seedlings of resistant and susceptible varieties, respectively. Significant differences in percentage share of time duration of specific EPG models during *R. padi* feeding upon the two varieties of maize (G-test, G = 69.04, df = 5) were ascertained. The results indicated that duration of pathway was the longest on both studied cultivars (38% and 46% of the probing time on the susceptible and resistant plants, respectively). The duration of phloem activities (E1 and E2) of *R. padi* was two times longer when feeding on the susceptible variety, compared to the resistant plants (33% and 17%, respectively). In addition, waveform pattern F, which reflects difficulties in stylet penetration throughout plant tissues, did not occur in any aphid recordings on the susceptible cultivar, but it was occasionally identified (0.02%) during feeding on the resistant seedlings.

## 3. Discussion

Plants exposed to adverse environmental stimuli respond by overproduction of ROS. It has been hypothesized that stress-induced ROS burst in plant tissues may be a causative agent of oxidatively-dependent damage to DNA, RNA, proteins, and lipids. Moreover, occurrence and severity of this oxidative damage are linked to the functioning of complex antioxidative machinery in the plant cells [15]. On one hand, an oxidative burst at the beginning of stress response comprises an element of the plant innate immunity, on the other hand, tolerance to the excessive ROS amounts is the key factor shaping plant growth and development under unfavorable conditions. Sytykiewicz [4] reported that *R. padi* infestation affected substantial overproduction of hydrogen peroxide (H_2_O_2_) at 24 hpi in Złota Karłowa and Waza seedlings. Furthermore, Sytykiewicz [11] uncovered that exogenous application of diphenyliodonium (DPJ), an inhibitor of NADPH oxidase, significantly lowered the activity of this enzyme as well as the accumulation of H_2_O_2_ in maize seedling leaves exposed to *R. padi* attack, indicating the crucial role of excessive formation of superoxide anion radicals in oxidative responses of maize seedlings.

Although oxidative stress-induced damage of DNA molecules has been revealed in some plant species in response to heavy metals, anthracene, and ozone treatments [16,17,18], there is no available information regarding the impact of biotic stressors on 8-hydroxy-2′-deoxyguanosine (8-OHdG) content in genomic DNA in plants. In the present study, we revealed only marginal and non-significant increases in the content of 8-OHdG in genomic DNA in aphid-stressed maize seedlings, compared to the uninfested control. Similarly, Bernard et al. [18] recorded insignificant elevations in the level of 8-OHdG in the leaves of forage kale (*Brassica oleracea* var. *viridis* cv. Prover) grown on a cadmium-contaminated field soil. However, Bidar et al. [17] elucidated that *Lolium perenne* L. and *Trifolium repens* L. plants grown on Cd-, Pb-, and Zn-contaminated soil, characterized with increased 8-OHdG content, dependent on metals’ uptake and bioaccumulation. In addition, Debiane et al. [16] demonstrated that anthracene-treated roots of chicory (*Cichorium intybus* L.) responded with about 2-fold increase in 8-OHdG level compared to the control. Furthermore, lower increases were found in the roots exposed to both anthracene and colonized with *Glomus intraradices* fungus that illustrated the protective role of endomycorrhization in diminishing the oxidative damages of the host plant’s DNA. Higher plants evolved sophisticated molecular mechanisms aimed at detecting and repairing DNA damages to a certain extent, in order to maintain its structural integrity and ensure functional stability under stressful conditions [19].

The current work demonstrated the accumulation of 8-hydroxyguanosine (8-OHG) in both total RNA and mRNA in aphid-infested maize Złota Karłowa and Waza seedlings, thus indicating the enhanced oxidation of these fractions of ribonucleic acid. It is the first report evaluating the scale and time-course alternations in RNA oxidation level in seedlings of aphid-resistant and aphid-susceptible maize cultivars. Insect-stressed Złota Karłowa seedlings exhibited approximately about 100% and 20% higher accumulation of 8-OHG in mRNA and total RNA, respectively, compared to Waza plants. In addition, the maximum level of RNA oxidation in insect-infested maize plants occurred at 24 hpi. Recently, Chmielowska-Bąk and colleagues [20] demonstrated that soybean seedlings exposed to cadmium stress had increased 8-OHG content in RNA molecules. Generally, high contents of 8-OHG were reported to be involved in alternations in genes’ expression and repressed synthesis of certain proteins [20,21,22,23]. Chmielowska-Bąk et al. [20] suggested that 8-OHG accumulation may be restricted to specific transcripts in a highly selective manner, but mode of action of this selective process is still poorly understood. Additionally, oxidative modifications in mRNAs were associated with mitigation of seed dormancy [21,22].

Elevated amounts of ROS (e.g., superoxide anion radicals) may initiate peroxidation reaction of plasma membrane lipids, especially polyunsaturated fatty acids (PUFAs). Malondialdehyde (MDA) is one of the major secondary products of lipid peroxidation of plasma membranes. Despite changes in the content of MDA and/or electrolyte leakage (EL) level in maize tissues in response to a wide range of abiotic stressors (e.g., drought, salinity, waterlogging, low/high temperature, exposure to heavy metals and xenobiotics) have been widely investigated [24,25,26,27], the impact of insect feeding on these oxidative stress markers was not studied in this context. In our study, aphid-induced elevation in MDA content and EL level in both tested maize cultivars was evident. However, the aphid infestation caused higher increases in the quantified markers of oxidative stress in susceptible Złota Karłowa seedlings in comparison with more resistant Waza plants, and the highest increment was noted at 24 hpi. Final products of lipid peroxidation are responsible for detrimental changes in physical properties of plasma membranes (e.g., destabilization of its permeability, decline in hydrophobic properties of the inner part of the membrane) [28]. Lipid peroxidation is also linked to inhibition of numerous biocatalysts and disturbances in functioning of transport proteins across the plasmalemma. It has also been reported that MDA may react with DNA or proteins, forming the respective adducts [29]. Detailed studies revealed that the electrolyte leakage from the plant cells was mainly associated with increased K^+^ efflux through the potassium channels [30]. This process was reported at the highest generation of ROS in response to stressful environmental conditions. Moreover, potassium depletion in the plant cells may enhance the activity of proteases and endonucleases that secondarily induce the programmed cell death.

Thiol compounds function as stress indicators and play a significant role in oxidative stress control as well as detoxification of xenobiotics what is related to the activity of hydrosulfide group [31]. The redox state of thiols plays a crucial role in determination of protein structure, regulation of enzymatic activity and control the activity of transcription factors [32,33]. In the current work, infestation of maize plants with *R. padi* females reduced level of total thiols (TT) and non-protein thiols (NPT) in host foliar tissues. Furthermore, the resistant cultivar responded a greater depletion in the content of TT. However, after 96 hpi, the susceptible plants characterized with increased TT and NPT contents, whereas Waza seedlings exhibited reduced level of these compounds at the end of the experiment. Many reports have focused on changes in the contents of TT and NPT under abiotic stress, but there are no studies concerning the role of these compounds in plant responses to biotic stress factors. Bhoomika et al. [34] observed no significant alterations in TT content in seedlings of rice cultivars (Al-sensitive and Al-tolerant) exposed to aluminum. According to these authors, the content of NPT increased in roots and shoots of the seedlings of Al-sensitive rice cultivar with increase in Al concentration. However, Al-tolerant seedlings increased the amount of NPT only at the initial phase of the experiment and increases were lower in comparison to those noted in Al-sensitive cultivar. Other results were obtained by Nagalakshmi and Prasad [35], where TT content in copper-treated *Scenedesmus bijugatus* Kützig cells remained almost stable, whereas NPT content decreased. The depletion of NPT level was also noted in *Z. mays* cultivars (Single Cross 122 and Single Cross 10) stressed by copper, Furthermore, the copper treatments resulted in a significant gradual increase in TT content in both studied cultivars [36]. Similar tendency was demonstrated by Kaur et al. [37], where the earthworm supplementation of cadmium-treated soils, increased the content of TT in *Brassica juncea* L. plants, whereas the amount of NPT was limited with increasing Cd concentration in soil. Similarly, high increases in the level of TT and NPT were observed in maize plants treated with isoproturon, but thiols were elevated only during the first few days and by low doses of the tested compound [38]. In contrast, the content of NPT in non-tolerant clone of *Chloris barbata* L. increased under cadmium stress [39].

Proteins are the most abundant cellular components vulnerable to ROS and constitute about 70% of the oxidized molecules in the cell [40]. Protein oxidation is one of the earliest responses of plants to stressors and is often used as a marker of oxidative stress [41]. Proteins may be oxidized in reversible and irreversible ways [42]. Thiol groups and sulphur containing amino acids are very susceptible to oxidation by ROS and are the most commonly modified [40,43]. The oxidation of protein cysteine thiol groups may generate thiyl radicals, disulfide bonds as well as sulfenic, sulfinic and sulfonic acid derivatives [42]. A decline in protein thiols reflects the oxidation of sulfhydryl groups of protein and is one of the indicators of oxidative stress [44]. The results of our study demonstrated more substantial reduction of PT content in resistant cultivar infested with *R. padi*. However, the short-time infestation (3 hpi) did not affect PT level in both studied maize cultivars. Opposite tendency was noted by Bhoomika et al. [34], where aluminum treatment profoundly reduced PT content in Al-sensitive rice cultivar, whereas the level of protein sulfhydryls in the seedlings of Al-tolerant cultivar remained unchanged. Authors speculated that the production of ROS and induction of oxidative stress by Al was greater in the seedlings of Al-sensitive cultivar than Al-tolerant one. On the contrary, we observed more marked elevation in superoxide anion radical (O_2_ **^•^**ˉ) amount in tissues of Ambrozja seedlings (relatively resistant maize cultivar) in comparison to Tasty Sweet (susceptible maize cultivar) infested with *R. padi* [6]. Additionally, Ambrozja seedlings stressed by *R. padi* females showed a more significant decrease in the total antioxidant capacity towards DPPH (1,1-diphenyl-2-picrylhydrazyl) radicals in relation to susceptible Tasty Sweet cultivar [5]. The depletion of protein-bound thiols was demonstrated in many plants subjected to abiotic stress factors. Namely, the significant depletion of protein sulfhydryls (about 19%) was noted in radish (*Raphanus sativus* L.) seedlings treated with zinc [44]. The salt stress caused a decrease in protein–SH in embryogenic suspension culture of *Dactylis glomerata* L. [45]. The dehydration resulted in a significant depletion of protein-bound thiols in *Triticum aestivum* L. seedlings; however, more sensitive plants exhibited a lower level of PT than tolerant ones [46]. The changes in the content of thiol compounds (*i.e*., TT, NPT and PT) in plants seem to be depended on the type of stress factor and intensity of oxidative stress. Some stressors (e.g., metals, herbicides) may induce the synthesis of TT or NPT, whereas biotic factors (e.g., aphids) caused depletion of these sulfhydryl compounds.

Another important oxidative damage of proteins is formation of the carbonyl derivatives. ROS may produce free carbonyl groups by reacting with amino acid side chains of protein molecules, particularly lysine, arginine, histidine, tryptophan and threonine residues [34]. Additionally, protein carbonylation may also be caused by protein reaction with lipid peroxidation products [41,47]. Protein carbonylation is an irreversible process and is used as a sensitive indicator of oxidative stress since the formation of protein carbonyls requires more stringent oxidation conditions than the oxidation of thiols [48,49,50]. We noted an increase in the content of protein carbonyls in maize seedlings after infestation with *R. padi*. Moreover, higher accumulation of protein-bound carbonyls occurred in tissues of susceptible Złota Karłowa cv. Plants differing in abiotic stress tolerance may exhibit diverse levels of protein oxidation. Drought-sensitive seedlings of *Oryza sativa* L. subjected to water-deficit, had a higher increase in protein carbonyl content, compared to the tolerant ones [51]. Roychoudhury et al. [52] proved that the carbonylated derivatives formation under cadmium treatment was greater in salt-sensitive variety of indica rice (IR-29) than in salt-tolerant variety (Nonabokra). The carbonyl groups content increased upon dehydration in wheat seedlings and the elevations were higher in sensitive seedlings in relation to tolerant ones [46]. Bhoomika et al. [34] recorded an increase in the protein carbonyl level after exposure of Al-sensitive rice cultivar to aluminum, but not in the Al-tolerant cultivar. Furthermore, exposure of maize seedlings to Pb enhanced the content of carbonyl groups (425–512%) during 3–24 h treatment [53]. Protein-bound carbonyl content increased in *Z. mays* varieties (Deccan and Sartaj) under chromium stress. The lower level of carbonylated protein was found in Deccan cv. which exhibited lower accumulation of O_2_**^•^**ˉ and H_2_O_2_ than Sartaj cv. [54]. Two lines of maize—Cat100-6 (Al-tolerant) and S1587-17 (Al-sensitive) were reported to exhibit different content of the carbonyl groups. The amount of carbonyls in Al-sensitive maize plants increased with an increase of Al concentration, but no changes were observed in the tolerant variety [55]. Authors suggested that differences in protein oxidation between two maize lines were related to the level of antioxidant defense. The results of our earlier studies showed that the most of the *sod* genes (encoding isoforms of superoxide dismutase) were more markedly upregulated in the aphid-resistant maize (Ambrozja cv.) than in the aphid-susceptible one (Tasty Sweet cv.) [5]. Furthermore, seedlings of aphid-infested resistant cultivar of *Z. mays* (Ambrozja) responded with higher levels in activity of ascorbate peroxidase (APX), monodehydroascorbate reductase (MDHAR), dehydroascorbate reductase (DHAR) and glutathione reductase (GR), in relation to susceptible cultivar (Tasty Sweet) [10]. Additionally, in the present study, the content of protein-bound carbonyls was even lower in tissues of control (non-infested) seedlings of resistant cultivar, which indicates the presence of more efficient antioxidant mechanisms or more effective system to remove of damaged proteins. The exposure of maize seedlings (Bosman cv.) to spider mite infestation caused an increase in protein carbonyl content coincided with the reduced catalase (CAT), polyphenol oxidase (PPO) and APX activities in comparison to the control plants. However, the combination of two stressors (mite feeding and drought), decreased the amount of carbonylated proteins despite the increased activity of all antioxidant enzymes (with exception of CAT) [56]. Authors postulated that protein carbonylation is not directly linked to oxidative stress based on the assessment of antioxidant enzyme activities, but may be a result of diminished capacity of oxidized protein removal or increased protein susceptibility to oxidative damages.

The current work revealed increases or fluctuations in levels of the quantified markers of oxidative damages of nucleic acids, proteins, and lipids in the control (aphid-free) maize seedlings (Figure 1, Figure 2, Figure 3, Figure 4, Figure 5, Figure 6, Figure 7 and Figure 8). These differences may be associated with growth of maize seedlings. At the two first time periods of the experiments (3 and 6 h), the seedlings were 14-day-old, whereas at the next time periods, they were 15-, 16- and 18-day-old, respectively. On the other hand, there is also a possibility that the control plants responded to volatiles emitted by the aphid-stressed maize plants. Based on these findings, further detailed studies are needed to uncover the mechanisms underlying changes in oxidative damages of the analyzed macromolecules in the control maize plants.

In the study, we found substantial differences in probing and feeding behavior of *R. padi* apterae within seedlings of Złota Karłowa cv., compared to Waza cv. The bird cherry-oat aphids fed longer on seedlings of susceptible cultivar than on resistant plants. We demonstrated that *R. padi* females ingested lower amounts of phloem sap while probing on Waza seedlings. The main decision of an aphid to accept or not a host plant occurs during the probing phase [57]. The probes vary in duration of time; however, short movement periods and prolonged probes are usually associated with good quality hosts. In general, the probes are shorter and aphids spend more time moving on the surface of the host when the ingested plant sap is characterized with a poor nutritive quality [58,59]. In the study, *R. padi* females performed first probes from few to a dozens of minutes after artificial attachment of the insects on seedling leaves of the studied maize cultivars, spending between 38% and 46% of the recorded time on penetration within epidermis and mesophyll tissues. Aphids uses their chemoreceptors to detect the presence and gradient of chemical compounds in host tissues. Longer time of penetration of aphid stylets in the epidermis and mesophyll and shorter or lack of contact with the sieve bundles may be caused by a higher concentration of allelocompounds in the resistant varieties. When feeding on more suitable host (susceptible Złota Karłowa cv.), *R. padi* apterous adults ingested quite large amounts of phloem sap. This led us to conclude that the resistance of Waza cv. is likely related to factors present in the phloem sap. The short phloem salivation phase on Waza seedlings indirectly indicated the presence of deterrent factors, that may impede aphids from acceptance of host plants [58,60]. In general, a higher concentration of allelochemicals may cause a reduction of the aphid feeding activities, corresponding to the duration of ingestion of the phloem sap. The rate of growth and reproduction of aphids depends on the quantity and quality of the consumed food (mainly the phloem sap). Similarly, Goławska et al. [59] and Kordan et al. [61] elucidated that that pea aphids (*Acyrthosiphon pisum* Harr.) spent less time on phloem sap ingestion on resistant cultivars of *Lupinus luteus* L., *Lupinus angustifolius* L. and *Medicago sativa* L. than on susceptible ones. Zehnder et al. [13] compared the probing behaviour of cowpea aphid (*Aphis craccivora* Koch) on susceptible and resistant varieties of yellow and narrow-leafed lupines, and they also observed that the aphids spent less time on phloem sap ingestion during infestation of resistant varieties, compared to susceptible ones.

## 4. Materials and Methods

### 4.1. Aphids

Wingless parthenogenetic females of the bird cherry-oat aphid (*Rhopalosiphum padi* L.) were collected from cereal crops in Siedlce district, Poland (52°09′54″ N, 22°16′17″ E). The insects were reared for a year on seedlings of common wheat (*Triticum aestivum* L., Tonacja cv.) in a climate chamber under specific conditions (photoperiod of L16:D8, temperature of 22 °C/16 °C (day/night), relative humidity of 70%, light intensity of 100 μM·m^−2^·s^−1^).

### 4.2. Plant Material

Seeds of two tested maize varieties (i.e., Waza and Złota Karłowa) were obtained from grain companies: PNOS S.A. (Ożarów Mazowiecki, Poland) and W. Legutko (Jutrosin, Poland). Based on previous experiments, Waza genotype was categorized as relatively resistant, whereas Złota Karłowa cultivar was susceptible to *R. padi* infestation [9]. All seeds were subjected to surface sterilization in 70% ethanol for 2 min, soaking in 0.1% HgCl_2_ for 3 min, and after that procedure, they were rinsed with deionized water. The seeds were planted separately in plastic pots (10 cm in diameter; 9 cm in height), filled with the universal garden soil (Kronen®; Lasland Sp. zo.o., Grądy, Poland). Maize seedlings were grown in the climatic chamber, under controlled conditions described above.

### 4.3. Leaf Infestation Experiments

Maize seedlings (14-day-old) of two tested varieties (i.e., Waza and Złota Karłowa) were infested with 30 or 60 apterous adult females of *R. padi* for 3, 6, 24, 48 and 96 h. Uninfested seedlings of a given maize cultivar were used as control. Only healthy seedlings of similar height were included in the experiments. Aphid-treated and control plants were caged in plastic transparent cylinders (20 cm in diameter; 50 cm in height), covered with one layer of the nylon mesh. In order to randomize the study, three independent series of the biotests were performed, and each treatment (0, 30, 60 aphids per plant) was replicated 30 times for each of the time points (*n* = 90). After termination of each series of the biotests, the insects were removed from the seedlings, and then, the leaf samples derived from 30 individual plants of each treatment combination were pooled and subjected to further biochemical analyses.

### 4.4. Extraction of Genomic DNA (gDNA)

Maize seedling leaves (50 mg portions) of each cultivar were harvested and immediately ground in liquid nitrogen. Isolation of gDNA from insect-infested and uninfested maize seedlings was performed with the use of Genomic Micro AX Plant Kit (A&A Biotechnology, Gdynia, Poland), following the manufacturer’s instructions. In addition, 5 mm^3^ of RNase (A&A Biotechnology) (stock concentration: 10 mg × cm^−3^) was added to gDNA samples in order to hydrolyse the residual amounts of RNA. The yield and purity of DNA samples were assessed with the use of Epoch UV-Vis microplate spectrophotometer (BioTek, Winooski, VT, USA).

### 4.5. Measurement of Oxidative Damages of Genomic DNA

Immunodetection of oxidative damages of gDNA molecules was achieved using EpiQuik™ 8-OHdG DNA Damage Quantification Direct Kit (Epigentek Group, Inc., Farmingdale, NY USA; catalogue no. P-6003-96), based on colorimetric (*λ* = 450 nm) quantitation of oxidized guanosine residues (8-hydroxy-2′-deoxyguanosine; 8-OHdG) in the tested maize samples. The input amount of 300 ng DNA was used per each assay reaction. The procedure was carried out according to the manufacturer’s instructions. The content of 8-OHdG in the maize samples was expressed in picograms per microgram of gDNA.

### 4.6. Isolation of Total RNA and mRNA

Total RNA and mRNA were extracted from aphid-infested and non-infested (control) maize seedlings. The freshly collected leaves were ground in liquid nitrogen with the use of sterile ceramic mortar and pestle. Isolation of total RNA from maize seedling leaves was carried out using Spectrum Plant Total RNA Kit (Sigma-Aldrich, Poznań, Poland; catalogue no. STRN50), and traces amount of genomic DNA was cleaved with On-Column DNase I Digestion Set (Sigma-Aldrich; catalogue no. DNASE70). Extraction of mRNA was achieved with application of Dynabeads mRNA DIRECT Purification Kit (Life Technologies, Warsaw, Poland; catalogue no. 61012). Isolation procedures were performed, according to the manufacturers’ protocols. Quantity and purity of RNA was evaluated using the Epoch UV-Vis microplate spectrophotometer (BioTek). Only intact and high-quality RNA samples were included in the study.

### 4.7. Determination of Oxidative Damages of Total RNA and mRNA

Estimation of oxidative damages of total RNA and mRNA was conducted using OxiSelect™ Oxidative RNA Damage ELISA Kit (Cell Biolabs, Inc., San Diego, CA, USA; catalogue no. STA-325). Quantitation of oxidized guanosine residues (8-hydroxyguanosine; 8-OHG) in RNA samples derived from maize foliar tissues was based on colorimetric measurements (*λ* = 450 nm). One microgram of the input amount of total RNA or mRNA per assay reaction was used. The procedure was performed following the manufacturer’s protocol. The content of 8-OHG in RNA samples was expressed in picograms per microgram of the analysed RNA fraction.

### 4.8. Determination of Total Thiols (TT), Non-Protein Thiols (NPT) and Protein Thiols (PT)

The contents of total thiols and protein thiols were determined according to Kok and Kuiper [62], with minor modifications. Fresh seedling leaves of *Z. mays* (600 mg) were ground to a fine powder in liquid nitrogen using pre-chilled mortar and pestle and extracted with ice-cold 0.2 M Tris-HCl buffer (pH 7.4). The extracts were centrifuged at 20,000× *g* for 10 min (4 °C). The supernatants were used for quantification of TT and NPT. In order to determine the total thiols, 0.5 cm^3^ of supernatant was mixed with 1 cm^3^ of 0.2 mM Tris-HCl (pH 8.2) and 0.1 cm^3^ of 0.01 M DTNB (5-5′-dithiobis(2-nitrobenzoic acid)). The reaction mixture was incubated at 30 °C for 15 min The yellow colour developed was measured at 415 nm. A correction was made for the absorbance of incubation mixture in the absence DTNB (replaced with deionized water) and in the absence of supernatant (replaced with 0.2 M Tris-HCl buffer; pH 7.4). During measurements the NPT content, the supernatant was deproteinized by incubating in a water bath at 100 °C for 5 min and centrifuged at 20,000× *g* for 10 min (4 °C). NPT content was determined in a similar manner to that used for the total thiols. The content of protein thiols was calculated by subtracting the content of non-protein thiols from the total thiols. Total and protein thiol groups were calculated from the extinction coefficient of 13,600 M^−1^ × cm^−1^. The content of thiols was expressed as micromoles per milligram of protein. The protein content in the analysed plant extracts was assayed using the method developed by Bradford [63].

### 4.9. Determination of Protein-Bound Carbonyls (CP)

The content of protein-bound carbonyls (CP) was determined according to the method of Levine et al. [64]. Fresh seedling leaves of *Z. mays* (600 mg) were ground to a powder in liquid nitrogen using pre-chilled mortar and pestle, and extracted with ice-cold 50 mM Na-phosphate buffer (pH 7.4) containing 1 mM EDTA. The homogenates were centrifuged at 6000× *g* for 10 min (4 °C). The supernatants were incubated on ice with 1% (*w/v*) streptomycin sulfate for 15 min and centrifuged at 6000× *g* for 10 min to remove the nucleic acid. Nucleic acid-free supernatants (200 mm^3^) were mixed with 800 mm^3^ of 10 mM 2,4-dinitrophenyl hydrazine (DNPH) in 2.5 M HCl. The blank samples were incubated in 2.5 M HCl. After 1 h incubation at ambient temperature (in the dark), 1 cm^3^ of 20% (*w/v*) TCA (trichloroacetic acid) was added, samples were incubated on ice for 5 min, and centrifuged at 10,000× *g* for 10 min The pellets were resuspended in 1 cm^3^ of ethanol: ethyl acetate (1:1) and centrifuged at 10,000× *g* for 10 min This procedure was repeated three times. The clean pellets were dissolved in 6 M guanidine hydrochloride in 20 mM K-phosphate buffer (pH 2.3), and centrifuged at 10,000× *g* for 10 min The absorbance was measured spectrophotometrically at 375 nm. Protein recovery was estimated for each sample by measuring the absorbance value at 280 nm. The carbonyl group content was calculated using a molar absorption coefficient for aliphatic hydrazones of 22,000 M^−1^ × cm^−1^ and expressed as nanomoles of carbonyls per milligram of protein.

### 4.10. Lipid Peroxidation Assay

Lipid peroxidation in the maize seedling leaves was based on measurement of malondialdehyde (MDA) content with the use of Lipid Peroxidation Assay Kit (Sigma-Aldrich; catalogue no. MAK085). Fresh leaf samples (10 mg) were ground in liquid nitrogen and homogenized with 300 mm^3^ of ice-cold MDA Lysis Buffer, containing 3 mm^3^ of butylated hydroxytoluene (BHT) solution. Next, the mixtures were centrifuged at 13,000× *g* for 10 min to remove insoluble material. The assay procedure was conducted following the manufacturer’s protocol. Lipid peroxidation level was based on reaction of MDA with thiobarbituric acid (TBA) to form a colorimetric (*λ* = 532 nm) product, proportional to the MDA amount present in the reaction mixture. The content of MDA in the maize samples was expressed in nanomoles of MDA per gram of fresh weight.

### 4.11. Plasma Membrane Stability Assay

Electrolytes’ leakage from the leaves of maize seedlings was estimated according to the conductometric method described by Jiang and Zhang [65]. The electrical conductivity was measured by using a pH/conductivity/salinity meter (model CPC-505; Elmetron, Zabrze, Poland). Percentage injury of plasma membranes at each aphid treatment was calculated from ion leakage data using the following equation: % membrane injuries (ion leakage) = [(%La − %Lc/(100 − %Lc)] × 100, where %La and %Lc are percentage ion leakage data for the aphid treatments and control samples, respectively.

### 4.12. Electrical Penetration Graph (EPG) Recordings

Feeding behaviour of adult apterous females of *R. padi* on 14-day-old maize seedlings (Waza and Złota Karłowa cvs) was monitored using the electrical penetration graph (EPG) technique, which is commonly applied in insect-plant relationship studies [66]. The experiments were run during 6 h for 20 randomly sampled aphids. Each aphid was monitored on individual maize seedlings, that were placed in plastic pots (10 cm in diameter; 9 cm in height), filled with the universal garden soil (Kronen®; Lasland Sp. z o.o., Grądy, Poland), and one plant per pot. The EPG tests were conducted in Faraday cage under the following conditions: temperature of 22 ± 1 °C, photoperiod of L16:D8, and relative humidity of 70%). In the present study, aphids were starved for 2 h prior to the experiment, and next, they were attached to a golden wire (20 μm in diameter and approximately 2–3 cm long), connected with the electrode with silver paint (L2027; Demetron, Darmstadt, Germany). Probing behaviour was monitored with a DC EPG amplifier (Giga 4 type). The activity of each aphid was recorded for 6 h by acquisition to a PC and analysed using STYLET 2.2 software (Wageningen University, Wageningen, The Netherlands). Typical waveforms patterns were investigated [67]. Duration time (min) of the following behavioural activities were determined: non-probing (Np pattern; aphids did not start probing), paths (C pattern; penetration of peripheral tissues—epidermis and mesophyll), sieve element salivation (E1 pattern), ingestion of phloem sap (E2 pattern—aphid feeding), mechanical movements of stylets associated with difficulties during tissue puncturing (F pattern) and xylem sap ingestion (G pattern).

### 4.13. Statistical Analyses

All data were calculated as the mean (±SEM) of at least three independent replicates. Three-factorial analysis of variance (ANOVA) was performed to examine the significance of the tested variables (i.e., maize genotype, number of aphids and infestation time), and the interactions between the studied variables on 8-OHdG content in gDNA, 8-OHG level in the tested RNA fractions, the content of protein-bound carbonyl groups, total thiols, non-protein and protein thiols, MDA and percentage of electrolyte leakage in aphid-challenged maize seedlings. Subsequently, post-hoc Tukey’s test was employed (*p* < 0.05 was set as significant). Significant differences for each EPG waveform between *R. padi* feeding activities on the relatively resistant and susceptible cultivars were estimated using Mann-Whitney U test (two samples test) at *p* < 0.05. The proportion of time devoted to different activities for *R. padi* on maize cultivars was compared using G test [20]. All analyses were conducted with the use of STATISTICA v.10 software (StatSoft Inc., Kraków, Poland).

## 5. Conclusions

In summary, *R. padi* feeding did not induce significant oxidative damages of genomic DNA in the host tissues, but considerable increases in the oxidation levels of mRNA and total RNA in aphid-infested maize plants were uncovered. In addition, higher oxidation of mRNA molecules in comparison with total RNA in insect-stressed maize seedlings was revealed. Bird cherry-oat aphid feeding led to a significant depletion in total, non-protein and protein thiols in maize seedlings. Moreover, the aphid herbivory can promote the formation of carbonyl groups in proteins, causing the irreversible damages in their structure. The resistant maize cultivar (Waza) characterized with a greater depletion in the thiols’ content, while the susceptible cultivar (Złota Karłowa) exhibited a higher accumulation of protein-bound carbonyls in response to the insect herbivory. Furthermore, aphid-stressed seedlings of susceptible cultivar had higher contents of MDA and level of electrolyte leakage, compared to relatively resistant plants. We also identified circumstantial differences in the stylet activities and feeding behavior of *R. padi* apterae within foliar tissues of Złota Karłowa cv. in relation to Waza plants. Collectively, higher degree of oxidative damages in RNA, proteins and lipids occurred in seedlings of the susceptible maize cultivar in comparison to the resistant variety. It indirectly proved that the antioxidant mechanisms in the aphid-resistant maize plants functioned more efficiently, in relation to the susceptible cultivar. Taking into consideration the importance of maize crops in agriculture, further investigations deciphering the signaling role of ROS-mediated damages of macromolecules in aphid-attacked host plants are needed.

## Figures and Tables

**Table 1:** Stylet activities of *R. padi* during feeding on the seedling leaves of the tested maize cultivars.

EPG Waveforms	EPG Activity			
	Number of Events	Total Duration (min)	Average Time (min)	Time to First Probing (min)
**Złota Karłowa cv. (susceptible)**				
Np	11.00 ± 4.71a	53.42 ± 15.02b	5.37 ± 2.13a	n/a
C	19.93 ± 6.91a	148.48 ± 62.01a	7.90 ± 3.48a	2.87 ± 1.77a
E1	9.33 ± 3.98a	26.16 ± 20.69a	2.80 ± 1.98a	37.07 ± 10.71a
E2	6.73 ± 3.22a	130.73 ± 75.24a	23.88 ± 19.44a	42.34 ± 11.22a
G	1.20 ± 1.78a	1.21 ± 128.62a	0.46 ± 0.96b	100.13 ± 142.93a
F	0.00 ± 0.00b	0.00 ± 0.00a	0.00 ± 0.00a	0.00 ± 0.00b
**Waza cv. (relatively resistant)**				
Np	12.80 ± 4.54a	143.31 ± 57.83a	12.53 ± 6.74a	n/a
C	19.27 ± 7.78a	152.04 ± 41.09a	8.52 ± 2.63a	4.77 ± 2.96a
E1	6.00 ± 4.05a	3.87 ± 3.39b	0.58 ± 0.26a	82.64 ± 17.28a
E2	5.00 ± 3.07a	22.65 ± 15.78b	4.21 ± 2.96b	83.32 ± 17.38a
G	2.93 ± 2.02a	37.66 ± 28.66a	11.92 ± 5.65a	81.23 ± 48.53a
F	1.07 ± 0.59a	0.47 ± 0.39a	0.38 ± 0.29a	88.64 ± 66.42a

Data are presented from 6-h EPG recordings (mean ± SEM; *n* = 20). Values for each EPG waveform within a column followed by different letters are significantly different between maize cultivars (Mann-Whitney U test; *p* < 0.05). Np—non-probing; C—transition of the insect stylets through epidermis and mesophyll cells; E1—salivation into sieve elements; E2—phloem sap ingestion; G—xylem sap uptake; F—difficulties in stylet penetrations in tissues of the host plant; n/a—not applicable.

## Supplementary Materials

Supplementary materials can be found at https://www.mdpi.com/1422-0067/20/15/3742/s1.
