# Supplementary material for: Aphid-Triggered Changes in Oxidative Damage Markers of Nucleic Acids, Proteins, and Lipids in Maize (Zea mays L.) Seedlings
Source: Int J Mol Sci. 2019 Jul 31;20(15):3742. doi: 10.3390/ijms20153742 (PMC6696134; doi:10.3390/ijms20153742)
Supplement: Supplementary file 1 [file ijms-20-03742-s001.pdf]

Table S1. Three-factorial ANOVA analysis of the examined variables (*i.e.*, maize genotype, number of aphids, infestation time) and the interactions on 8-OHdG content in genomic DNA, 8-OHG content in total RNA and mRNA, amount of MDA and percentage of electrolyte leakage (EL) in aphid-challenged maize seedlings.

**Indicators**	**8-OHdG (DNA)**	**8-OHG (Total RNA)**	**8-OHG (mRNA)**	**MDA**	**EL**
Maize genotype (G)	$F_{1, 54}$ = 12 (ns)	$F_{1, 54}$ = 2,682 (***)	$F_{1, 54}$ = 6,542 (***)	$F_{1, 54}$ = 2,375 (***)	$F_{1, 54}$ = 1,815 (***)
Number of aphids (A)	$F_{2, 54}$ = 4 (ns)	$F_{2, 54}$ = 1,945 (***)	$F_{2, 54}$ = 4,744 (***)	$F_{2, 54}$ = 2,060 (***)	$F_{2, 54}$ = 1,618 (***)
Infestation time (T)	$F_{5, 54}$ = 6 (ns)	$F_{5, 54}$ = 1,308 (***)	$F_{5, 54}$ = 1,630 (***)	$F_{5, 54}$ = 1,530 (***)	$F_{5, 54}$ = 934 (***)
G × A	$F_{2, 54}$ = 1 (ns)	$F_{2, 54}$ = 953 (***)	$F_{2, 54}$ = 804 (**)	$F_{2, 54}$ = 815 (***)	$F_{2, 54}$ = 675 (***)
A × T	$F_{10, 54}$ = 2 (ns)	$F_{10, 54}$ = 680 (**)	$F_{10, 54}$ = 915 (***)	$F_{10, 54}$ = 550 (***)	$F_{10, 54}$ = 411 (**)
G × T	$F_{5, 54}$ = 0.8 (ns)	$F_{5, 54}$ = 192 (**)	$F_{5, 54}$ = 457 (**)	$F_{5, 54}$ = 165 (*)	$F_{5, 54}$ = 98 (*)
G × A × T	$F_{10, 54}$ = 0.3 (ns)	$F_{10, 54}$ = 106 (*)	$F_{10, 54}$ = 235 (**)	$F_{10, 54}$ = 86 (*)	$F_{10, 54}$ = 52 (*)

(*) $P<0.05$; (**) $P<0.01$; (***) $P<0.001$; (ns) – non-significant. Variables: i) maize genotype – Waza and Złota Karłowa; ii) number of aphids – 0, 30 and 60 *R. padi* females per plant; iii) infestation time – 0, 3, 6, 24, 48 and 96 hours post infestation.

Table S2. Three-factorial ANOVA analysis of the studied variables (*i.e.*, maize genotype, number of aphids, infestation time) and the interactions on the content of total thiols, protein and non-protein thiols, and protein-bound carbonyl groups in aphid-stressed maize seedlings.

**Indicators**	**Total thiols**	**Protein thiols**	**Non-protein thiols**	**Protein carbonyls**
Maize genotype (G)	$F_{1, 54}$ = 3,965 (***)	$F_{1, 54}$ = 4,450 (***)	$F_{1, 54}$ = 6,385 (***)	$F_{1, 54}$ = 1,145 (***)
Number of aphids (A)	$F_{2, 54}$ = 2,613 (***)	$F_{2, 54}$ = 3,825 (***)	$F_{2, 54}$ = 2,374 (***)	$F_{2, 54}$ = 902 (***)
Infestation time (T)	$F_{5, 54}$ = 2,420 (***)	$F_{5, 54}$ = 1,690 (***)	$F_{5, 54}$ = 3,068 (***)	$F_{5, 54}$ = 1,347 (***)
G × A	$F_{2, 54}$ = 1,078 (***)	$F_{2, 54}$ = 945 (**)	$F_{2, 54}$ = 1,280 (***)	$F_{2, 54}$ = 615 (**)
A × T	$F_{10, 54}$ = 714 (**)	$F_{10, 54}$ = 816 (**)	$F_{10, 54}$ = 984 (***)	$F_{10, 54}$ = 264 (**)
G × T	$F_{5, 54}$ = 180 (*)	$F_{5, 54}$ = 205 (*)	$F_{5, 54}$ = 317 (**)	$F_{5, 54}$ = 396 (**)
G × A × T	$F_{10, 54}$ = 163 (*)	$F_{10, 54}$ = 158 (*)	$F_{10, 54}$ = 195 (**)	$F_{10, 54}$ = 112 (*)

(*) $P<0.05$; (**) $P<0.01$; (***) $P<0.001$; (ns) – non-significant. Variables: i) maize genotype – Waza and Złota Karłowa; ii) number of aphids – 0, 30 and 60 *R. padi* females per plant; iii) infestation time – 0, 3, 6, 24, 48 and 96 hours post infestation.
